# Safety and efficacy of different tirofiban administration routes on acute ischemic stroke patients with successful recanalization: A propensity score matching analysis

**DOI:** 10.1111/cns.13936

**Published:** 2022-08-13

**Authors:** Wenting Guo, Jiali Xu, Linqing Ma, Jin Ma, Sijie Li, Changhong Ren, Longfei Wu, Chuanjie Wu, Chuanhui Li, Jian Chen, Jiangang Duan, Qingfeng Ma, Haiqing Song, Wenbo Zhao, Xunming Ji

**Affiliations:** ^1^ Department of Neurology, Xuanwu Hospital Capital Medical University Beijing China; ^2^ Department of Neurology The People's Hospital of Suzhou New District Suzhou China; ^3^ Beijing Key Laboratory of Hypoxic Conditioning Translational Medicine, Xuanwu Hospital Capital Medical University Beijing China; ^4^ Department of Emergency, Xuanwu Hospital Capital Medical University Beijing China; ^5^ Department of Neurosurgery, Xuanwu Hospital Capital Medical University Beijing China; ^6^ Beijing Institute of Brain Disorders Capital Medical University Beijing China

**Keywords:** prognosis, stroke, thrombectomy, tirofiban

## Abstract

**Objective:**

This study aimed to explore the effect of different administration routes of a low dose of tirofiban on acute ischemic stroke (AIS) patients with successful recanalization after endovascular treatment (EVT).

**Methods:**

This is a cohort study that retrospectively analyzed data of patients with AIS who underwent EVT and achieved successful recanalization from a prospective registry. Eligible patients were divided into three groups according to their use of tirofiban. Propensity score matching (PSM) was used to balance baseline bias. Safety outcomes included any intracranial hemorrhage (ICH) and symptomatic ICH (sICH). Efficacy outcomes included arterial reocclusion, in‐hospital mortality, 3‐month mortality, and 3‐month functional outcomes.

**Results:**

We included 821 patients with 306 in the no tirofiban group, 202 in the IA + IV tirofiban group, and 313 in the IV tirofiban group. After PSM, each group included 101 patients with balanced baseline characteristics. There was no difference between the IV tirofiban group and the no tirofiban group in terms of safety and efficacy outcomes (all *p* > 0.05). Compared with no tirofiban, IA + IV tirofiban group did not increase ICH (30.7% vs. 37.6%, *p* > 0.05) and sICH (6.9% vs. 17.8%, *p* > 0.05) whereas reduced 3‐month mortality (14.3% vs. 28.7%, *p* < 0.05) and improved 3‐month modified Rankin Scale (median 3 vs. 4, *p* < 0.05).

**Conclusions:**

A low dose of tirofiban, regardless of their administration routes, was safe for AIS patients who achieved successful recanalization with EVT, whereas only IA + IV tirofiban improved clinical outcomes.

## Graphical abstract

This study found that a low dose of intra‐arterial + intravenous tirofiban improved 3‐month clinical outcomes (reduced mortality and improved functional outcomes) of acute ischemic stroke patients with successful recanalization by endovascular treatment, while intravenous tirofiban did not.

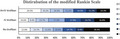

## 1 INTRODUCTION

Acute ischemic stroke (AIS) is a severe and life‐threatening disease, especially for those secondary to large‐vessel occlusions. Intravenous thrombolysis (IVT) with alteplase and endovascular treatment (EVT) are the only two evidence‐based treatment options available for AIS currently.^1^ Compared with IVT, EVT was much more effective in recanalizing the proximal occlusions of intracranial arteries and improving the clinical outcomes of patients.^2^ It has been reported that EVT could yield a successful recanalization rate of around 80% or more compared with traditional therapies.^2^, ^3^, ^4^ However, about 40%–50% of the successfully recanalized patients still experienced unfavorable outcomes at 3 months.^5^, ^6^ The gap between successful recanalization and unfavorable outcomes may be partly attributed to the thromboembolic complications and early arterial reocclusion caused by endothelial damage, plaque disruption, platelet activation, and subsequent platelet aggregation.^7^ Therefore, great attention has been attracted to explore safe and effective therapies to prevent platelet aggregation perioperatively with the purpose of improving clinical prognoses after successful recanalization.

Glycoprotein IIb/IIIa receptor, exclusively expressed on the membranes of platelets and megakaryocytes, can be activated by adenosine diphosphate, epinephrine, collagen, or thrombin to bind fibrinogen, thereby bridging platelets together to induce platelet aggregation.^8^, ^9^ Tirofiban is a highly selective glycoprotein IIb/IIIa receptor antagonist, which can effectively block the final pathway of platelet aggregation and subsequent thrombus formation, thereby providing potential benefits to successfully recanalized patients with AIS. Recently, its safety and efficacy for AIS patients who underwent EVT have been explored in a series of observational clinical studies; however, conflicting results were reported.^10^, ^11^, ^12^, ^13^, ^14^, ^15^, ^16^ These controversial findings might be attributed to the heterogeneity of the administration routes of tirofiban and the study population.^17^ However, few studies discussed the effect of different administration routes of tirofiban on clinical outcomes of patients with EVT, especially for patients with successful recanalization. Therefore, to explore whether tirofiban can serve as an effective adjunct therapy and its optimal protocol for successfully recanalized AIS patients, our study compared the safety and efficacy of different administration routes of tirofiban (intra‐arterial [IA] + intravenous [IV] tirofiban vs. IV tirofiban vs. no tirofiban) for AIS patients who achieved successful recanalization with EVT.

## 2 METHODS

### 2.1 Study population

This study retrospectively selected patients from a prospective cohort that recorded data of consecutive AIS patients who underwent EVT at Xuanwu Hospital between January 2013 and June 2021, the inclusion criteria were as follows: (1) age ≥18 years, (2) pretreatment modified Rankin Scale (mRS) ≤2, (3) EVT performed within 24 h, (4) successful recanalization defined as the modified thrombolysis in cerebral infarction (mTICI) scale of 2b or 3, and (5) available complete baseline data. This prospective cohort had approval from the Ethics Committee of Xuanwu Hospital. Written informed consent was obtained from each patient. All EVT procedures were performed following the recommendation of current guidelines.^1^

### 2.2 Data collection

Variables collected from the database included age, sex, vascular risk factors, prestroke drug use, baseline characteristics, lesion site, stroke etiology, treatment information, and safety and efficacy outcomes. Baseline characteristics included admission systolic blood pressure (SBP), diastolic blood pressure (DBP), stroke severity assessed using the National Institute of Health Stroke Scale (NIHSS) and the Alberta Stroke Program Early Computed Tomography Score (ASPECTS) or posterior circulation ASPECTS (pc‐ASPECTS). Stroke etiology was classified according to the Trial of Org 10,172 in Acute Stroke Treatment (TOAST). Treatment information included IVT, anesthesia mode, time interval from symptoms onset to groin puncture (OTP), time interval from symptoms onset to recanalization (OTR), EVT details, and tirofiban administration routes.

### 2.3 Intervention of tirofiban

The decision on tirofiban treatment and its administration route was at the discretion of interventionists. In general, tirofiban was considered as the following cases: (1) patients receiving rescue treatment with emergency stenting or balloon angioplasty for failed thrombectomy; (2) successful recanalization by three or more passes with a stent retriever with a high potential for endothelial injuries; (3) severe in situ atherosclerosis with a high risk of early reocclusion; and (4) no indications for intracranial hemorrhage (ICH) on instant computer tomography (CT).

Patients in this study were divided into three groups according to the tirofiban administration route: the no tirofiban group, the IA + IV tirofiban group, and the IV tirofiban group. Namely, tirofiban was routinely injected with a bolus dose of 0.25–0.5 mg intra‐arterially or intravenously first, followed by an intravenous infusion of 0.2–0.4 mg/h for 12–24 h. Besides, all enrolled patients were treated following the standard protocol recommended by the current guideline.^1^

### 2.4 Safety and efficacy outcomes

The safety outcomes included any ICH and symptomatic ICH (sICH). sICH is defined according to the European Cooperative Acute Stroke Study III before discharge.^18^ The efficacy endpoints included the following: (1) early reocclusion of the recanalized artery assessed using transcranial Doppler or CT angiography, (2) in‐hospital mortality, (3) mortality at 3 months, (4) mRS score at 3 months, and (5) favorable outcome defined as a mRS score of 0–2 at 3 months. All of the safety and efficacy outcomes were evaluated blindly by qualified neurologists and trained staff. Patients with missing data on safety and efficacy outcomes were excluded from their corresponding analysis.

### 2.5 Statistical analysis

All data were analyzed using SPSS (Version 26.0; IBM Corp) and R Project for Statistical Computing (Version 4.1.2). *p*‐value < 0.05 (two‐sided) was considered significant. Patients in our study were divided into three groups, and differences in baseline characteristics of the three groups were compared. Kolmogorov–Smirnov tests are used to assess the distribution of continuous data. Normality data are expressed as mean ± standard deviation and tested using the one‐way analysis of variance (ANOVA) for their differences across the three groups. Nonnormality data are expressed as median (interquartile) and tested using the Kruskal–Wallis test for their differences across the three groups. Categorical data are summarized using numbers (percentages) and tested using the chi‐square test.

Propensity score matching (PSM) is applied to match subjects with a similar distribution of confounders to achieve an estimation of treatment effects with minimal bias, and it is more advantageous for observational studies to use PSM controlling confounders compared with traditional regression methods, especially in the condition that the number of confounders is large, or the number of outcomes is limited. Therefore, PSM was used to balance the baseline covariates and reduce data bias. Confounders selected in this study must not be influenced by tirofiban which also should be measured before tirofiban is given. Baseline variates with statistically significant differences among the three groups will be matched. Using the TriMatch package of the R statistical software, patients in the three groups were matched at a 1:1:1 ratio according to their baseline characteristics using PSM analysis that applied the nearest‐neighbor matching with a caliper of the distance of 0.1 combined with the exact matching of lesion site and TOAST. After PSM, comparison of baseline characteristics and outcomes of the three groups were analyzed again. Pairwise comparisons of the three groups were conducted with Bonferroni post hoc tests.

## 3 RESULTS

**FIGURE 1:**
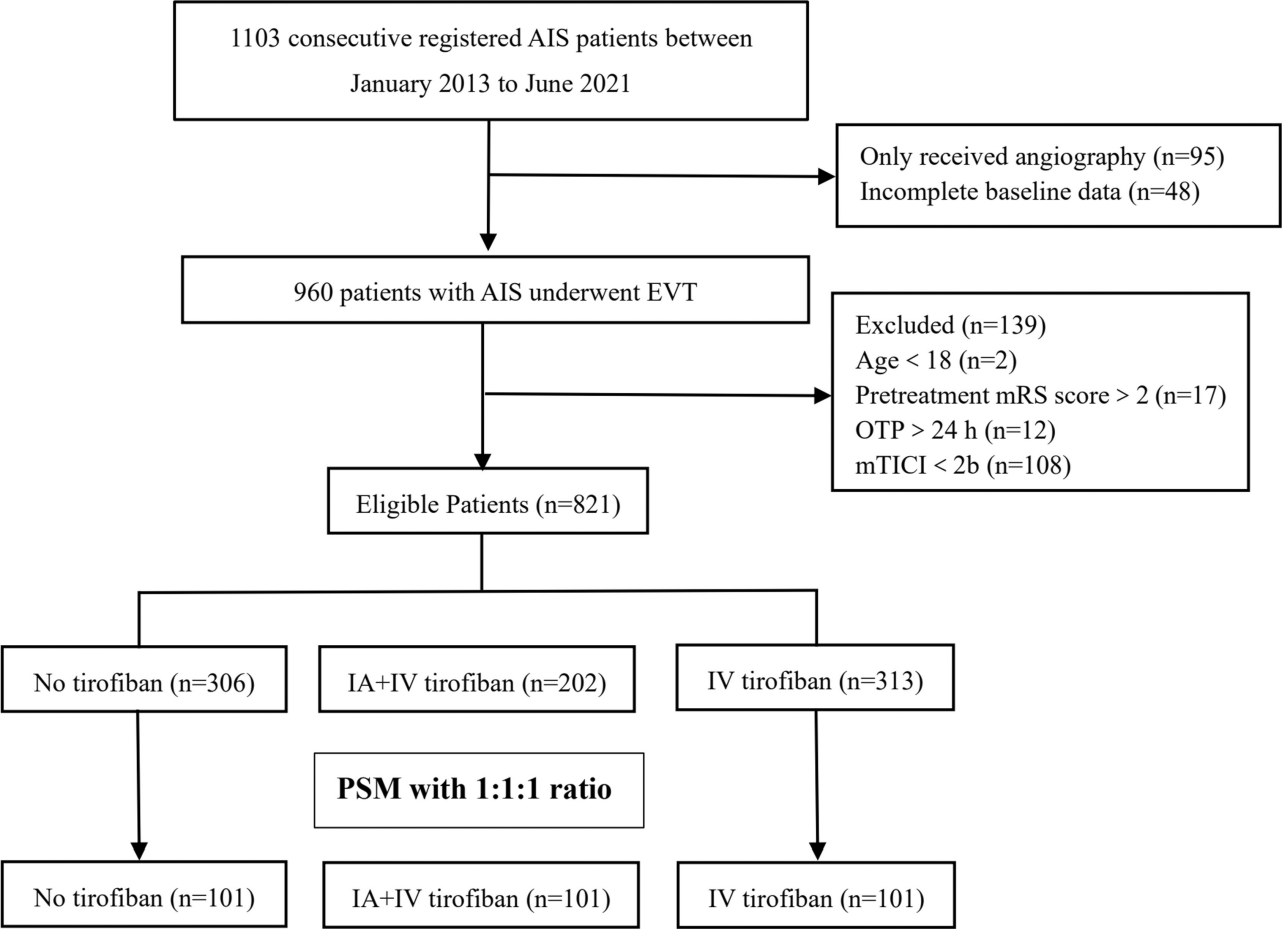
Flow chart. AIS, acute ischemic stroke; EVT, endovascular treatment; mRS, modified Rankin Scale; OTP, time interval from symptoms onset to puncture; mTICI, modified thrombolysis in cerebral infarction

Between January 2013 and June 2021, a total of 1103 patients were registered and screened. Ninety‐five patients were excluded for they only received angiography, and 48 were excluded for incomplete baseline characteristics. Therefore, there were 960 patients underwent EVT at Xuanwu Hospital (2 with age <18 years old, 17 with previous mRS score >2, 12 with OTP >24 h, 108 with mTICI <2b). Finally, this study included 821 AIS patients who underwent EVT and achieved successful recanalization (mean age: 62.9 ± 12.2 years, 71.4% of male), of whom 306 patients were in the no tirofiban group, 202 patients were in the IA + IV tirofiban group, and 313 patients were in the IV tirofiban group (Figure 1).

### 3.1 Baseline characteristics of patients before and after PSM

Baseline characteristics of subjects in different tirofiban groups were summarized in Table 1. We found that age, sex, hypertension, current smoking, current drinking, atrial fibrillation, previous anticoagulation, lesion site, stroke etiology, general anesthesia, OTP, OTR, additional intra‐arterial thrombolysis, and stent retriever were significantly different among three groups (*p* < 0.05). The PSM resulted in 101 matched triplets with balanced baseline characteristics. The comparison of baseline characteristics between the three groups after PSM is shown in Table 2. These matched 101 triples had no missing data for baseline variables. Finally, 288 (95%) patients completed the 3‐month follow‐up.

**TABLE 1:** Baseline characteristics of patients before PSM

Variables (Before PSM)	No Tirofiban (*n* = 306)	IA + IV Tirofiban (*n* = 202)	IV Tirofiban (*n* = 313)	*p*‐Value
Demography				
Age (year), mean (SD)	64.7 ± 13.3	60.3 ± 10.9	62.9 ± 11.5	**<0.001** *
Male, *n* (%)	187 (61.1%)	155 (76.7%)	244 (78%)	**<0.001** *
Vascular risk factors				
Hypertension, *n* (%)	202 (66%)	156 (77.2%)	218 (69.6%)	**0.025** *
Diabetes, *n* (%)	78 (25.5%)	60 (29.7%)	98 (31.3%)	0.262
Hyperlipidemia, *n* (%)	169 (55.2%)	110 (54.5%)	191 (61%)	0.226
Current smoking, *n* (%)	98 (32%)	90 (44.6%)	137 (43.8%)	**0.003** *
Current drinking, *n* (%)	77 (25.2%)	83 (41.1%)	108 (34.5%)	**0.001** *
Atrial fibrillation, *n* (%)	147 (48%)	32 (15.8%)	85 (27.2%)	**<0.001** *
Previous stroke, *n* (%)	80 (26.1%)	52 (25.7%)	79 (25.2%)	0.967
Drug use prestroke				
Antiplatelet, *n* (%)	92 (30.1%)	59 (29.2%)	102 (32.6%)	0.675
Anticoagulation, *n* (%)	31 (10.1%)	6 (3.0%)	22 (7.0%)	**0.009**
Baseline characteristics				
SBP (mmHg), mean (SD)	145.9 ± 23.5	149.7 ± 24.9	148.1 ± 22.9	0.193
DBP (mmHg), mean (SD)	82.8 ± 15.4	84.8 ± 14.0	85.6 ± 14.5	0.056
NIHSS, median (IQR)	16 (9)	16 (14)	15 (8)	0.124
ASPECTS/pc‐ASPECTS, median (IQR)	9(3)	9 (2)	9 (3)	0.758
Lesion site				
Anterior circulation, *n* (%)	240 (78.4%)	114 (56.4%)	229 (73.2%)	**<0.001** *
Posterior circulation, *n* (%)	66 (21.6%)	88 (43.6%)	84 (26.8%)	
Stroke etiology				
LAA, *n* (%)	119 (38.9%)	166 (82.2%)	211 (67.4%)	**<0.001** *
CE, *n* (%)	169 (55.2%)	29 (14.4%)	90 (28.8%)	
Others, *n* (%)	18 (5.9%)	7 (3.5%)	12 (3.8%)	
Treatment information				
IVT, *n* (%)	117 (38.2%)	59 (29.2%)	100 (31.9%)	0.079
General anesthesia, *n* (%)	119 (38.9%)	98 (48.5%)	91 (29.1%)	**<0.001** *
OTP (min), median (IQR)	346 (188)	376 (235)	432 (289)	**<0.001** *
OTR (min), median (IQR)	420 (187)	478 (236)	498 (282)	**<0.001** *
Additional intra‐arterial thrombolysis, *n* (%)	14 (4.6%)	17 (8.4%)	11 (3.5%)	**0.041** *
Aspiration, *n* (%)	159 (52%)	104 (51.5%)	171 (54.6%)	0.724
Stent, *n* (%)	241 (78.8%)	160 (79.2%)	215 (68.7%)	**0.004** *

Abbreviations: PSM, pronspensity score matching; IA, intra‐arterial; IV, intravenous; ASPECTS, alberta stroke program early computed tomography score; CE, cardioembolism; DBP, diastolic blood pressure; IVT, intravenous thrombolysis; LAA, large artery atherosclerosis; NIHSS, national institute of health stroke scale; OTP, time interval from symptoms onset to puncture; OTR, time interval from symptoms onset to recanalization; pc‐ASPECTS, posterior circulation alberta stroke program early computed tomography score; SBP, systolic blood pressure.

Comparison of baseline characteristics of patients in the no tirofiban, IA+IV tirofiban, and IV tirofiban groups before PSM.

* *p* < 0.05.

**TABLE 2:** Baseline characteristics of patients after PSM

Variables (After PSM)	No Tirofiban (*n* = 101)	IA + IV Tirofiban (*n* = 101)	IV Tirofiban (*n* = 101)	*p*‐Value
Demography				
Age (year), mean (SD)	61.7 ± 12.5	60.7 ± 10.8	60.2 ± 12.1	0.666
Male, *n* (%)	81 (80.2%)	78 (77.2%)	79 (78.2%)	0.872
Vascular risk factors				
Hypertension, *n* (%)	74 (73.3%)	75 (74.3%)	80 (79.2%)	0.574
Diabetes, *n* (%)	33 (32.7%)	25 (24.8%)	37 (36.6%)	0.180
Hyperlipidemia, *n* (%)	60 (59.4%)	59 (58.4%)	65 (64.4%)	0.651
Current smoking, *n* (%)	41 (40.6%)	45 (44.6%)	49 (48.5%)	0.527
Current drinking, *n* (%)	34 (33.7%)	39 (38.6%)	39 (38.6%)	0.702
Atrial fibrillation, *n* (%)	22 (21.8%)	21 (20.8%)	23 (22.8%)	0.944
Previous stroke, *n* (%)	32 (31.7%)	24 (23.8%)	22 (21.8%)	0.235
Drug use prestroke				
Antiplatelet, *n* (%)	33 (32.7%)	29 (28.7%)	23 (22.8%)	0.289
Anticoagulation, *n* (%)	7 (6.9%)	4 (4.0%)	11 (10.9%)	0.163
Baseline characteristics				
SBP (mmHg), mean (SD)	149.2 ± 22.3	148.1 ± 22.8	152.8 ± 23.2	0.478
DBP (mmHg), mean (SD)	85.3 ± 15.9	88.9 ± 13.8	84.5 ± 13.3	0.068
NIHSS, median (IQR)	16 (10)	16 (15)	17 (10)	0.887
ASPECTS/pc‐ASPECTS, median (IQR)	9 (3)	9 (2)	9 (3)	0.378
Lesion site				
Anterior circulation, *n* (%)	71 (70.3%)	71 (70.3%)	71 (70.3%)	1.000
Posterior circulation, *n* (%)	30 (29.7%)	30 (29.7%)	30 (29.7%)	
Stroke etiology				
LAA, *n* (%)	76 (72.5%)	76 (75.2%)	76 (75.2%)	1.000
CE, *n* (%)	24 (23.8%)	24 (23.8%)	24 (23.8%)	
Others, *n* (%)	1 (1.0%)	1 (1.0%)	1 (1.0%)	
Treatment information				
IVT, *n* (%)	32 (31.7%)	31 (30.7%)	35 (34.7%)	0.822
General anesthesia, *n* (%)	42 (41.6%)	43 (42.6%)	42 (41.6%)	0.987
OTP (min), median (IQR)	382 (194)	368 (230)	420 (385)	0.186
OTR (min), median (IQR)	470 (234)	463 (211)	490 (496)	0.445
Additional intra‐arterial thrombolysis, *n* (%)	5 (5.0%)	3 (3.0%)	3 (3.0%)	0.686
Aspiration, *n* (%)	46 (45.5%)	48 (47.5%)	52 (51.5%)	0.691
Stent, *n* (%)	77 (76.2%)	85 (84.2%)	75 (74.3%)	0.196

Abbreviations: PSM, pronspensity score matching; IA, intra‐arterial; IV, intravenous; ASPECTS, alberta stroke program early computed tomography score; CE, cardioembolism; DBP, diastolic blood pressure; IVT, intravenous thrombolysis; LAA, large artery atherosclerosis; NIHSS, national institute of health stroke scale; OTP, time interval from symptoms onset to puncture; OTR, time interval from symptoms onset to recanalization; pc‐ASPECTS, posterior circulation alberta stroke program early computed tomography score; SBP, systolic blood pressure.

Comparison of baseline characteristics of patients in the no tirofiban, IA+IV tirofiban, and IV tirofiban groups after PSM.

### 3.2 Safety outcomes

Table 3 displayed the safety and efficacy outcomes of patients after PSM. For the safety outcomes, 38 patients (37.6%) in the no tirofiban group experienced ICH, 31 patients (30.7%) in the IA + IV tirofiban group experienced ICH, and 30 patients (29.7%) in the IV tirofiban group experienced ICH. There was no significant difference in ICH among the three groups (*p* = 0.425). The proportion of sICH was different in the IA + IV tirofiban group, the IV tirofiban group, and the no tirofiban group (6.9% vs. 6.9% vs. 17.8%, *p* = 0.015), despite no significant difference achieved in pairwise comparisons.

**TABLE 3:** Outcomes of patients after PSM (101)

Outcomes	No Tirofiban	IA + IV Tirofiban	IV Tirofiban	*p*‐Value
Safety outcomes				
Any ICH, *n* (%)	38(37.6%) ^a^	31(30.7%) ^a^	30(29.7%) ^a^	0.425
sICH, *n* (%)	18(17.8%) ^a^	7(6.9%) ^a^	7(6.9%) ^a^	**0.015**
Efficacy outcomes				
Reocclusion, *n* (%) *	10(10.6%) ^a^	10(10.0%) ^a^	13(13.7%) ^a^	0.692
In‐hospital mortality, *n* (%)	15(14.9%) ^a^	5(5.0%) ^a^	5(5.0%) ^a^	**0.013**
3‐month mortality, *n* (%) ^@^	27(28.4%) ^a^	14(14.3%) ^b^	16(16.8%) ^a, b^	**0.033**
3‐month mRS, median (IQR) ^@^	4(5) ^a^	3(4) ^b^	3(3) ^a^	**0.039**
3‐month mRS 0–2, *n* (%) ^@^	36(37.9%) ^a^	48(49%) ^a^	31(32.6%) ^a^	0.060

*Note*: *Data of reocclusion were available for 289 of 303 patients with 94 in the no tirofiban group, 100 in the IA + IV tirofiban group, and 95 in the IV tirofiban group. ^@^Data of clinical outcomes at 3 months were available for 288 of 303 patients with 95 in the no tirofiban group, 98 in the IA + IV tirofiban group, and 95 in the IV tirofiban group. Pairwise comparisons among the three groups are shown using superscripts of ^a^ and ^b^. Same superscripts mean no difference between two groups, while different superscripts mean a significant difference between two groups.

### 3.3 Efficacy outcomes

Data of reocclusion were available for 289 of 303 patients with 94 in the no tirofiban group, 100 in the IA + IV tirofiban group, and 95 in the IV tirofiban group. There was no significant difference in the proportion of reocclusion among the three groups after PSM (10.6% vs. 10.0% vs. 13.7%, *p* = 0.692). In‐hospital mortality was different in the three groups with 14.9%, 5.0%, and 5.0% in the no tirofiban group, IA + IV tirofiban group, and IV tirofiban group, respectively (*p* = 0.013). However, there was no significant difference in pairwise comparisons of the in‐hospital mortality.

**FIGURE 2:**
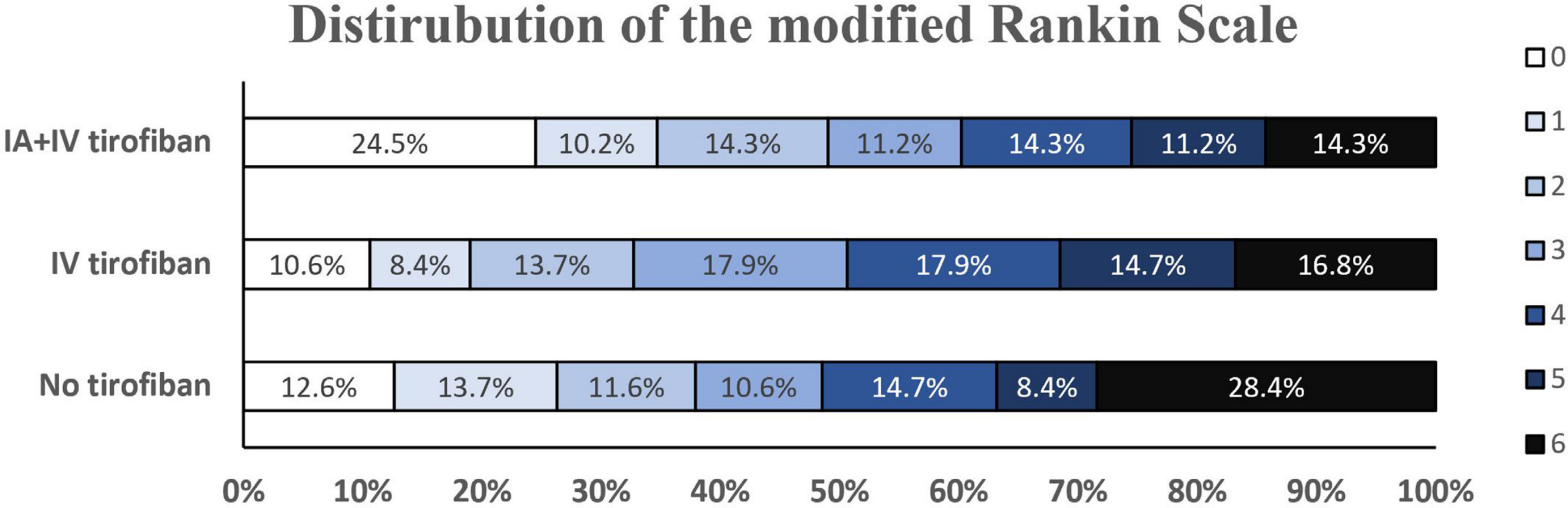
Distribution of the modified Rankin Scale at 3 month among the three groups

Data of clinical outcomes at 3 months were available for 288 of 303 patients with 95 in the no tirofiban group, 98 in the IA + IV tirofiban group, and 95 in the IV tirofiban group. The mortality at 3 month was 28.4%, 16.8%, and 14.3% in the no tirofiban group, IV tirofiban group, and IA + IV tirofiban with a significant difference among them (*p* = 0.033, Table 3, Figure 2). The pairwise comparison showed that 3‐month mortality in the IA + IV tirofiban group was significantly lower than 3‐month mortality in the no tirofiban group. However, no significant difference was found in mortality at 3 months between the no tirofiban group and the IV tirofiban group. The median mRS score at 3 months was 4 (IQR: 5), 3 (IQR: 4), and 3 (IQR: 3) in patients who receive no tirofiban, IA + IV tirofiban, and IV tirofiban with a significant difference among them (*p* = 0.039, Table 3, Figure 2). The pairwise comparison showed that patients receiving IA + IV tirofiban had a significantly better mRS score at 3 months than that the patients receiving no tirofiban. However, no significant difference was found between the no tirofiban group and the IV tirofiban group. The proportion of favorable outcomes seems to be more in the IA + IV tirofiban group (49%) than in the no tirofiban group (37.9%) and the IV tirofiban group (32.6%), despite no significant difference being found among them (*p* = 0.060).

## 4 DISCUSSION

Our study found that (1) neither a low dose of IA + IV tirofiban nor IV tirofiban increased the risk of ICH and sICH in patients with AIS who underwent EVT and received successful recanalization; and (2) a low dose of IA + IV tirofiban was associated with a decreased mortality and an improved functional outcome at 3 months.

As one of the antiplatelet drugs, the safety and efficacy of tirofiban as an adjunctive treatment to EVT have been discussed in recent years. However, conflicting findings were reported. Several studies found that tirofiban did not improve clinical outcomes and increased ICH risk in AIS patients treated with EVT. However, others showed that tirofiban was not associated with higher ICH and tended to lower mortality and improve functional outcomes.^12^, ^15^, ^19^, ^20^ The discrepancy in results among these studies may be due to the various dose and administration routes of tirofiban. Therefore, the optimal protocol of tirofiban as an adjunct to patients receiving EVT remains to be explored.

Wu et al found a dose‐dependent effect of IA tirofiban on ICH and sICH.^16^ He proposed that a low dose of IA tirofiban might be safer as an adjunctive therapy for AIS patients receiving EVT compared with a high dose of IA tirofiban. Many other studies also showed that a low dose of IA tirofiban was a safe adjunctive therapy for patients with EVT compared with no tirofiban.^12^, ^15^, ^21^, ^22^ Regarding IV tirofiban, its safety compared with no tirofiban has also been confirmed by several previous studies.^10^, ^11^, ^14^, ^23^ However, scarce studies compared the effect of IA + IV tirofiban with IV tirofiban. The only study by Yang et al.^17^ reported that a full dose of IA tirofiban (10 μg/Kg) followed by continuous IV tirofiban (0.15 μg/Kg) brings a higher hemorrhagic risk compared with a full dose of IV tirofiban or no tirofiban. Our study is the first to compare the safety and efficacy of different injective routes of a low dose of tirofiban, and we found that a lower dose of tirofiban was safe enough for successfully recanalized patients with AIS regardless of their administration routes. Intriguingly, our results also showed a trend of a lower sICH rate in patients with IA + IV tirofiban or IV tirofiban compared with no tirofiban. This phenomenon might be explained for the following reasons (1) tirofiban was more likely to be used in patients with a low risk of sICH at the discretion of interventionists, and (2) more attention was paid to patients receiving tirofiban to avoid their hemorrhagic risk.

Whether various administration routes could affect the efficacy of tirofiban remains unknown. We found that a low dose of IA + IV tirofiban brought significant beneficial effects (reducing mortality and improving functional outcomes at 3 months) to AIS patients with successful recanalization compared with no tirofiban, which was consistent with previous studies.^12^, ^15^, ^21^, ^22^ However, the novel finding was that a low dose of IV tirofiban was insufficient to improve clinical outcomes in our study. We presumed that IA bolus injection might quickly increase local drug concentration and provide direct contact of tirofiban to the thrombus to effectively inhibit subsequent platelet aggregation compared with IV bolus injection, thereby exerting its beneficial effects. Although several previous studies reported IV tirofiban improved functional outcomes at 3 months, which seemed to be contrary to our results, a relatively high dose of tirofiban used in their studies was an unignorable factor that may lead to the discrepancy.^11^, ^17^, ^24^ Therefore, we speculated that IV tirofiban might be administrated with a high dose to exert its maximal effects.

The beneficial effects of IA + IV tirofiban in our study may not be associated with the decreased reocclusion rate after EVT, which is consistent with previous studies that reported patients receiving tirofiban had similar reocclusion rates compared with patients not receiving tirofiban.^25^, ^26^ However, Yan et al.^22^ reported that the use of a low dose of IA + IV tirofiban was associated with both a lower reocclusion rate and favorable outcomes for large artery atherosclerotic occlusion stroke patients with residual stenosis after EVT. Heterogeneity in the study population may account for this discrepancy. Given that the prevention of large‐vessel reocclusion was not depicted in our study, we supposed the benefits of IA + IV tirofiban may be related to keeping and improving microvascular patency for successfully recanalized patients due to the effect of tirofiban in blocking activated platelet aggregation and subsequent thrombus formation.

This study has some limitations. First, the results of this study were based on an observational study that included subjects from only one region of China, which would have introduced selection bias. Second, the decision on tirofiban treatment and its administration route was determined by interventionists, potential confounders related to clinical outcomes might not be balanced despite PSM analysis being used to reduce their impact. What is more, we presumed that both a low dose of IA + IV tirofiban and a high dose of IV tirofiban were beneficial to successfully recanalized AIS patients by EVT, but we can not compare the effect of low dose IA + IV with high‐dose IV tirofiban due to the limitation of our data. Further exploration using multicenter randomized clinical trials is warranted to substantiate our findings.

## 5 CONCLUSION

In conclusion, in patients with AIS who underwent EVT and achieved successful recanalization, neither a low dose of IA + IV tirofiban nor IV tirofiban was associated with an increased risk of ICH and sICH. What is more, a low dose of IA + IV tirofiban leads to lower mortality and improved functional outcomes at 3 month. Further studies are warranted to confirm our findings and determine the optimal tirofiban protocol.

## FUNDING INFORMATION

This study was supported by Beijing Nova Program (No. Z201100006820143), National Natural Science Foundation of China (No. 82001257, 81801313, and 81971114) and General Project of Science and Technology of Beijing Municipal Education Commission (No. KM202110025018).

## CONFLICT OF INTEREST

The authors declare no conflict of interest.

## CONSENT TO PARTICIPATE

Written informed consent was obtained from all patients or their legal representatives.

## Data Availability

The data supporting the findings of this study are available from the corresponding author on reasonable request.
